# Conditioning generative latent optimization for sparse-view computed tomography image reconstruction

**DOI:** 10.1117/1.JMI.12.2.024004

**Published:** 2025-04-01

**Authors:** Thomas Braure, Delphine Lazaro, David Hateau, Vincent Brandon, Kévin Ginsburger

**Affiliations:** a CEA DIF, Arpajon Cedex, France; b Université Paris-Saclay, CEA, List, Palaiseau, France

**Keywords:** sparse-view computed tomography, unsupervised deep learning, latent optimization, convolutional neural network, image reconstruction

## Abstract

**Purpose:**

The issue of delivered doses during computed tomography (CT) scans encouraged sparser sets of X-ray projection, severely degrading reconstructions from conventional methods. Although most deep learning approaches benefit from large supervised datasets, they cannot generalize to new acquisition protocols (geometry, source/detector specifications). To address this issue, we developed a method working without training data and independently of experimental setups. In addition, our model may be initialized on small unsupervised datasets to enhance reconstructions.

**Approach:**

We propose a conditioned generative latent optimization (cGLO) in which a decoder reconstructs multiple slices simultaneously with a shared objective. It is tested on full-dose sparse-view CT for varying projection sets: (a) without training data against Deep Image Prior (DIP) and (b) with training datasets of multiple sizes against state-of-the-art score-based generative models (SGMs). Peak signal-to-noise ratio (PSNR) and structural SIMilarity (SSIM) metrics are used to quantify reconstruction quality.

**Results:**

cGLO demonstrates better SSIM than SGMs (between +0.034 and +0.139) and has an increasing advantage for smaller datasets reaching a +6.06  dB PSNR gain. Our strategy also outperforms DIP with at least a +1.52  dB PSNR advantage and peaks at +3.15  dB with fewer angles. Moreover, cGLO does not create artifacts or structural deformations contrary to DIP and SGMs.

**Conclusions:**

We propose a parsimonious and robust reconstruction technique offering similar to better performances when compared with state-of-the-art methods regarding full-dose sparse-view CT. Our strategy could be readily applied to any imaging reconstruction task without any assumption about the acquisition protocol or the quantity of available data.

## 1 Introduction

Imaging inverse problems (IIPs) are a large and swiftly growing area of research. IIPs cover a large variety of applications, ranging from generic image processing topics such as denoising, super-resolution, and deconvolution to more specific domains usually involving the characterization of a system of interest from indirect observations. In this latter case, the problem of reconstructing scans from medical imaging devices such as computed tomography (CT), magnetic resonance imaging (MRI), and positron emission tomography (PET) offers major challenges yet to be solved. Among those, CT reconstruction is a prime example of medical IIPs. Developments to reduce delivered doses to patients have resulted in sparser sets of X-ray projection angles and/or a decrease in X-ray intensities resulting in noisier, less contrasted, and detailed projection images. When using such degraded measurement setups, reconstructions obtained by conventional numerical methods, e.g., filtered back-projection (FBP),^1^ are severely worsened.

Recent progress has been made using deep learning approaches for CT reconstruction. However, a large part of these novel data-driven methods employ a supervised training setup.^2^^–^^8^ In supervised training pairs, ground truths consist of 3D volumetric scans reconstructed from conventional techniques such as the well-known FBP algorithm, using high-dose and densely sampled X-ray measurements. A degraded set of measurements is associated with each ground truth. These are usually obtained by taking a subset of the original X-ray projections (full-dose sparse view CT or sparse CT) and/or by adding noise to simulate dose reduction (low-dose CT). Building such supervised datasets thus implies that a fixed physical model has been employed for all training pairs, which must be identical to the one used for inference. In other words, supervised reconstruction strategies need new training whenever the acquisition process changes. This can be problematic when the viewing angles, X-ray spectrum, or beam geometry vary. To circumvent this drawback, several unsupervised strategies have recently been introduced.

Current approaches dealing with ill-posed IIPs in an unsupervised way are mostly based on the use of generative models. As such, the generative adversarial networks (GANs)^9^ are largely employed for unsupervised reconstruction tasks.^10^ As serious contenders to GANs, diffusion models (DMs)^11^^,^^12^ have gained a lot of attention in the past few years, as recent improvements have led to the generation of high-quality image samples without requiring a complex adversarial optimization.^13^^–^^16^ The basic principle of DMs is to add noise of increasing intensity to data during the training process and learn to reverse this process, i.e., starting from random noise and producing a sample from the data distribution through successive denoising. In the continuous approach,^17^ the discrete sequence of noise levels is replaced by a continuum of distributions progressively diffusing data points into random noise following a stochastic differential equation (SDE) describing a Markovian diffusion process. The reverse-time SDE is approximated with a time-dependent neural network estimating the gradient of the prior likelihood, also called the score function. Using standard iterative sampling techniques then leads to generative capability. Following this general strategy, two types of SDE formulations can be distinguished: the variance-preserving denoising diffusion probabilistic models (DDPMs)^12^^,^^18^ and the variance-exploding score-matching generative models (SGMs).^11^^,^^17^^,^^19^ In particular, recent works involving DMs have proven strong performances in solving IIPs^20^^–^^24^ and more specifically, sparse CT reconstruction.^23^^,^^25^^,^^26^ Both GANs and DMs are very efficient in learning unconditional prior distributions on slices of 3D reconstructed volumes. However, difficulties arise when a conditional sampling is needed to reconstruct slices from experimental X-ray projections. Various strategies have been proposed to deal with this conditional sampling issue.

GAN inversion approaches^10^ start from a trained and fixed decoder and aim at estimating the latent code corresponding to a given observation. The inversion process can either be learning-based,^27^^,^^28^ e.g., learning an encoder to inverse the decoder, or optimization-based where the optimization problem is solved by finding the latent code minimizing the given objective function,^29^^,^^30^ or both.^31^^–^^33^ Other approaches using a parameterization of the latent space have been proposed, where the decoder and the encoder are jointly learned using an adversarial process.^34^^–^^40^ Similar to GANs, conditional inference using DMs is an open field of research. It is generally performed using a biased sampling of the posterior distribution to generate approximate samples from a stochastic process conditioned to experimental measurements. For instance, in the context of sparse CT reconstruction, conditioning an SGM (cSGM) to X-ray projections, as in Ref. 25, involves computing the FBP on experimental measurements completed with simulated measurements from the candidate reconstruction, i.e., a proximal projection step throwing the sample path off the data manifold according to Ref. 26. In the latter, a manifold constraint on the gradient (MCG) is enforced during the sampling process as an additional correction term resulting in more robust reconstructions. Although both these methods are restricted to linear IIPs, a recent work from Chung et al.,^23^ known as deep posterior sampling (DPS), has extended the use cases of DMs to noisy nonlinear problems. As interestingly noted in Ref. 23, DPS is equivalent to MCG in noiseless configurations, e.g., full-dose sparse view CT, which is the application of interest in this paper.

Unsupervised generative models require large amounts of training data to have good generalization properties, but in practice, it is not clear how much is necessary. Indeed, in the literature, most generative models are tested in asymptotic conditions regarding data availability and training time. The behavior of these models with limited training data is hardly discussed. However, the size of training datasets may be limited to accommodate targeted studies regarding a combination of specific modalities, demographics, pathologies, and/or expert labelings. Furthermore, for some modalities (e.g., MRI with contrast agent), collecting a large dataset may be too time-consuming or too costly for a small organization. Finally, training computation on a larger dataset may be a restricting factor given limited access to advanced hardware.

To address this issue, Ulyanov et al. proposed a method specific to IIP solving, Deep Image Prior (DIP).^41^ They showed that the structure of a deep convolutional network inherently captures a significant amount of low-level image statistics without any learning. That is to say, it induces an implicit structural bias, i.e., a prior. DIP uses a randomly initialized U-Net architecture^42^ and a fixed input noise. The network weights can then be optimized to solve any ill-posed IIP with a known forward model, such as inpainting or super-resolution. It has been shown that this IIP reparameterization, when optimized with gradient descent, hierarchically reconstructs from low to high frequencies, i.e., converging faster toward “natural images” than noise.^43^^,^^44^ The method has recently been adapted in Ref. 45 to solve low-dose and sparse CT reconstruction. Although this strategy showed good results on use cases with missing or small datasets, it imposes a quite rough prior, which cannot compete with the reconstruction performances of its data-driven counterparts in degraded measurement setups.

This paper bridges the gap between, on one side, data-hungry generative models offering strong priors, and data-free methods providing weak priors on the other side, by proposing a reconstruction method readily usable with or without training data. Our method is a conditional version of the Generative Latent Optimization (GLO)^46^ framework. GLO was designed as a generative model attempting to produce competitive results when compared with GANs without using an adversarial training protocol. Models building on GLO are focused on improving its generative characteristics,^47^^–^^49^ and to the best of our knowledge, it has never been conditioned to solve IIPs. Indeed, exploring or sampling the latent space of GLO is not trivial,^48^^,^^49^ thus making it difficult to apply standard GAN inversion technique to directly solve IIPs. Instead, in this work, GLO is used to reparameterize the IIP. Similarly to DIP, our conditioned version of GLO (cGLO) exploits the inherent structural prior induced by a convolutional decoder, allowing reconstructions without any training dataset. However, contrary to DIP, cGLO can benefit from a shared objective by reconstructing multiple slices at the same time and an unsupervised training dataset of any size to initialize its decoder weights, resulting in greatly improved reconstruction results. The higher the quantity of training data used for initialization, the stronger the induced prior is.

The paper is organized as follows. Section 2 details our cGLO method, built upon the reparameterization strategy of DIP and the framework of GLO. Consequently, both DIP and GLO are introduced beforehand. Section 3 describes the experimental setups used to benchmark our method on the full-dose sparse view CT reconstruction task. These setups correspond to situations with sparse or abundant training data. Then, implementation details regarding network architecture and hyperparameter values are given. The section ends with a discussion on computational complexity. Our method cGLO is then compared, in Sec. 4, through quantitative and qualitative results for two distinct scenarios. It is first compared without any training data available to DIP, FBP, and a standard regularized least squares with a total variation (TV) iterative reconstruction technique designated as TV-Reg. Then, it is compared with cSGM and MCG with prior unsupervised training on datasets of varying sizes. The paper concludes with a summary and suggestions for future works in Sec. 5.

## 2 Method

The method we propose here builds on the framework used by GLO and on the DIP approach, which is a method dedicated to solving IIPs through reparameterization of the objective. This reformulation of DIP is detailed for a denoising task and for its adaptation to sparse CT reconstruction. Then, GLO and the concept of latent optimization are presented in the context of unconditioned generation, i.e., not directly applicable to IIP solving. Finally, our method cGLO is described with an objective function corresponding to the experiments conducted in this paper, i.e., full-dose sparse CT.

### 2.1 Deep Image Prior

Ulyanov et al.^41^ introduced DIP to solve classic IIPs such as denoising and super-resolution. The core idea of DIP is to regularize IIPs by taking advantage of the structural bias of a U-Net^42^ $f_{\theta }$ parameterized with a set of weights θ. It operates through two mechanisms: reparameterization and early stopping (ES). For instance, in the case of denoising, the optimization problem is reparameterized as

$$x^{*}=\{f_{\theta^{*}}(z)|\theta^{*}=\underset{\theta }{\mathrm{argmin}}\;{\left\|f_{\theta }(z)-(x_{0}+\eta )\right\|}_{2}^{2}\},$$ (1)

where $x_{0}$ is the initial image perturbed with unknown white noise η and z is a fixed white noise with the same dimensions as $x_{0}$. Experiments in Ref. 41 showed that given sufficient capacity and time/iterations, via gradient descent, the randomly initialized and over-parameterized U-Net can fit the output signal almost perfectly, including the noise η. However, Ulyanov et al.^41^ showed that the weights descent sequence $\theta_{1},\ldots ,\theta_{N}$, with $f_{\theta_{N}}\approx x_{0}+\eta$, contains an early stopping point $\theta_{\mathrm{ES}}$, such that $f_{\theta_{\mathrm{ES}}}\approx x_{0}$. This phenomenon has been proven to be a consequence of the structure of the generative network, more specifically the convolutional and upsampling layers.^43^^,^^44^ They induce a spectral bias in which the decoder learns to construct the image from low to high frequencies, meaning that with an appropriate choice of ES, one can prevent the decoder from fitting the high-frequency perturbations.

This methodology has recently been tailored by Baguer et al.^45^ to estimate 2D CT reconstructions of patient slices. To that end, an adaptation of Eq. (1) is used. It is further regularized with TV, i.e., the L1-norm of the gradient

$$x^{*}=\{f_{\theta^{*}}(z)|\theta^{*}=\underset{\theta }{\mathrm{argmin}}\;{\left\|T_{\phi_{e}}\circ f_{\theta }(z)-y_{\phi_{e}}\right\|}_{2}^{2}+\alpha \mathrm{TV}\circ f_{\theta }(z)\},$$ (2)

where $T_{\phi_{e}}$ is the Radon transform sampled on experimental viewing angles $\phi_{e}$, $y_{\phi_{e}}$ are the experimental measurements, and α is an hyper-parameter balancing the regularization. In this formulation, the issue of finding an appropriate early stopping for denoising is replaced with the necessity to find an optimal value for α, depending on the ill-posedness of the IIP at hand.

Further experiments in Ref. 45 showed improvements by initializing the decoder weights using the result $x_{s}$ of a supervised method, e.g., learned primal-dual reconstruction^4^

$$\theta_{0}=\underset{\theta }{\mathrm{argmin}}\;{\left\|f_{\theta }(z)-x_{s}\right\|}_{2}^{2}.$$ (3)

However, replacing random weights of the decoder with such a supervised initialization restricts the applicability of the approach to the supervised use case, i.e., fixed acquisition parameters. To avoid this, Baguer et al. used for this initialization a reconstruction computed with standard data-free techniques instead, e.g., FBP.

### 2.2 Generative Latent Optimization

The GLO technique uses a decoder network and a set of learnable unit noise vectors, i.e., latent codes, where each code is associated with a single individual of the unsupervised training dataset. During training, the gradient is back-propagated both on the weights of the decoder and on the latent codes.

Formally it consists of mapping one freely learned unit latent vector $z_{i}$ to each image $x_{i}$ of a given dataset $X=\{x_{1},\ldots ,x_{N}\}$, through a decoder network $f_{\theta }$ with parameters θ, e.g., a DCGAN.^50^ With gradient descent, it comes to jointly learn decoder network parameters and latent codes $Z=\{z_{1},\ldots ,z_{N}\}$, both randomly initialized, with the objective function

$$Z^{*},\theta^{*}=\underset{Z,\theta }{\mathrm{argmin}}\;\frac{1}{N}\overset{N}{\underset{i=1}{\sum }}\|f_{\theta }(z_{i})-x_{i}{\|}_{2}^{2}\;\mathrm{s.t.}\;{\left\|z_{i}\right\|}_{2}=1.$$ (4)

It is mentioned in Ref. 46 that, as is conventionally done for training GANs, vectors sampled from an n-dimensional normal distribution are close to the surface of an n-sphere with radius $\sqrt{n}$. Following this idea, Bojanowski et al. proposed to constrain the latent vectors to the unit n-sphere. It facilitates the exploration of the latent space and allows data transformation via geodesic interpolation. This constraint does not deteriorate the optimization and is numerically simple as it consists of projecting the latent vectors onto the unit n-sphere, i.e., normalizing, after each back-propagation.

Experiments in Refs. 46 and 47 have shown that the GLO approach does not suffer from mode collapse and significantly outperforms GANs when trained on small datasets. However, such as variational auto-encoders (VAE),^51^ the quality of generated samples deteriorates when the data variability overcomes the network capacity.^52^

### 2.3 Conditioning GLO

Following GAN inversion literature,^10^ one could try to search the latent space to find the best candidate latent vector so that the output of the decoder is close to experimental observations. However, attempting to invert GLO, i.e., sampling or exploring the manifold entailed by latent vectors learned through Eq. (4), is not straightforward.^48^^,^^49^ The latent vectors are not uniformly distributed on the surface of the n-sphere. Furthermore, the decoder generation quality rapidly worsens when evaluated on vectors outside geodesic interpolation lines between pairs of latent vectors. In other words, the manifold on which the trained decoder can produce plausible images can be described as a complete graph, heterogeneously distributed on the surface of the unit n-sphere.

Instead of inverting GLO, our method cGLO uses a reparameterization of the IIP, such as DIP,^41^ to benefit from the structural bias of the convolutional decoder. However, the objective function described in Eq. (2), is cast into the framework of GLO,^46^ detailed in Eq. (4).

At examination time, without prior training, the K slices $\overset{¯}{X}=\{{\overset{¯}{x}}_{1},\ldots ,{\overset{¯}{x}}_{K}\}$ from one or several patients are reconstructed together such that the parameters θ, and the latent unit vectors $\overset{¯}{Z}=\{{\overset{¯}{z}}_{1},\ldots ,{\overset{¯}{z}}_{K}\}$ are jointly learned through

$${\overset{¯}{Z}}^{*},\theta^{*}=\underset{\overset{¯}{Z},\theta }{\mathrm{argmin}}\;\frac{1}{K}\overset{K}{\underset{i=1}{\sum }}{\left\|T_{\phi_{e}}\circ f_{\theta }({\overset{¯}{z}}_{i})-y_{i,\phi_{e}}\right\|}_{2}^{2}\;\mathrm{s.t.}\;\|{\overset{¯}{z}}_{i}{\|}_{2}=1,$$ (5)

$${\overset{¯}{X}}^{*}=f_{\theta^{*}}({\overset{¯}{Z}}^{*}),$$ (6)

where $T_{\phi_{e}}$ is the Radon transform sampled on experimental viewing angles $\phi_{e}$ and $y_{i,\;\phi_{e}}$ are the experimental profiles obtained from slice ${\bar{x}}_{i}$. This method comes with two improvements: (i) it avoids the overfitting issue of DIP because of the shared decoder between the entire set of slices ${\bar{X}}^{*}$, given sufficient slices to reconstruct, and (ii) leads to better reconstructions by adding supplementary experimental profiles to the objective tends to stabilize the optimization process detailed in Eq. (5). Furthermore, the set of profiles can easily be augmented by linear interpolation along the vertical axis, i.e., the axis orthogonal to the slices plane. In this case, reconstructions are conducted using the entire augmented set, then reconstructions corresponding to interpolated measurements are discarded. The impact of the number of profiles, K, on performances is detailed in the ablation study in Appendix A.

Given the availability of a training set of full-dose CT scans, i.e., a set of slices $X=\{x_{1},\ldots ,x_{N}\}$, additional prior knowledge may be enforced by learning an appropriate initial set of parameters $\theta_{0}$, for the decoder $f_{\theta }$, in an unsupervised manner following Eqs. (3) and (4):

$$\theta_{0}=\underset{Z,\theta }{\mathrm{argmin}}\;\frac{1}{N}\overset{N}{\underset{i=1}{\sum }}\|f_{\theta }(z_{i})-x_{i}{\|}_{2}^{2}\;\mathrm{s.t.}\;\|z_{i}{\|}_{2}=1.$$ (7)

It is expected for the set of parameters $\theta_{0}$ to be closer to $\theta^{*}$ than random weights, thus easing the optimization of Eq. (5), especially when $\phi_{e}$ is very sparse. The latent vectors from Eq. (7) are not reused so that the set $\bar{Z}$ of Eq. (5) is always randomly initialized.

## 3 Experiments

Experiments have been conducted on two commonly used datasets, which are publicly available on The Cancer Imaging Archive (TCIA) platform.^53^ The methods involved in these experiments are (a) cGLO, TV-Reg, FBP,^1^ and DIP^45^ without training data and (b) cGLO, cSGM,^25^ and MCG^26^ with training sub-datasets of varying sizes. In their original papers, MCG and cSGM are trained on the complete LIDC/LDCT datasets. After training these models with the same architecture, number of parameters, and learning conditions as in their original designs, we found that similar performances were obtained with only 35% portions of the datasets. Training on larger portions did not achieve significant performance gain. Consequently, sub-datasets are limited to 35% portions of the initial datasets. All the methods are compared with the same number of experimental viewing angles: 9, 23, and 50. These numbers of projections are similar to those used in cSGM and MCG articles. This range allows for an accurate estimation of the effect of the number of viewing angles on the tested approaches. Restricting experiments to a higher number of projections would result in smaller differences between model, which might fall within the margin of error and results in a less conclusive comparison. The DPS method^23^ is omitted as it is equivalent to MCG for experiments conducted in this work, i.e., full-dose sparse CT. Implementation details concerning our decoder network architecture and hyperparameter values for each method are provided at the end of the section.

### 3.1 Datasets

The Lung Image Database Consortium (LIDC)^54^ image collection was collected using lung cancer screenings from 1018 patients. It consists of thoracic CT scans completed with radiologists’ annotations for nodule segmentation. The low-dose CT image and projection dataset (LDCT)^55^ is comprised of 299 CT scans of patient heads, chests, and abdomens and their respective (full) clinical doses. In addition, the LDCT dataset also includes simulated reduced doses, by Poisson noise insertion, and the location and diagnosis for positive findings. In the presented experiments, only CT scan slices from both datasets are used. The native resolution of slices is 512×512  pixels for both datasets, but the LIDC slices are downsized to a resolution of 320×320  pixels. For experiments to be representative of various realistic situations with sparse or abundant data, training sub-datasets consisting of portions of the LIDC and LDCT collections are prepared. Details of such datasets are given in Table 1. The LIDC and the LDCT test sets consist of five scans respectively totaling 791 and 1089 slices. Regarding experiments involving training sub-datasets, peak signal-to-noise ratio (PSNR) and structural SIMilarity (SSIM) metrics are estimated on the total test set where each 3D volume is reconstructed independently. It means that five independent reconstructions are computed, one for each scan in the test set. Each reconstruction estimates all the slices of a single scan, following a typical CT workflow. However, experiments conducted with cGLO and DIP, without any training datasets, are tested on a restricted set of 100 slices from one patient due to the computational time of the DIP approach. For these latter experiments, reconstructions computed with FBP and associated quantitative metrics are added as a baseline.

**Table 1:** Sub-datasets used for training with their corresponding number of 3D scans and slices. 3D scans are always fully included.

Portion	# of scans	# of slices
(a) LIDC sub-datasets		
35%	350	65 033
10%	100	17 898
2%	20	2 906
(b) LDCT sub-datasets		
35%	103	16 924
10%	29	4 989
2%	6	930

### 3.2 Forward Operator

During the training on sub-datasets, generated slices are directly compared through a pixel-wise metric to pre-computed reconstructions available in the datasets, e.g., in Eq. (7). As shown in Eq. (5), during the reconstruction process, estimated slices are indirectly compared with ground-truth profiles via projections with a discrete forward operator $T_{\phi_{e}}$ given the set of angles $\phi_{e}$. For every experiment conducted in this paper, the same discrete forward operator is used for the simulation of ground-truth profiles $y_{\phi_{e}}$ from slices in LIDC and LDCT test sets, and for the simulated projections in the reconstruction process. The discrete simulation of projections follows a parallel-beam geometry with equidistant angles between 0 deg and 180 deg, and equidistant detector bins spanning the slice diagonal, i.e., 453 for LIDC ($320\sqrt{2}$) and 725 for LDCT ($512\sqrt{2}$).

### 3.3 Implementation Details

**Fig. 1:**
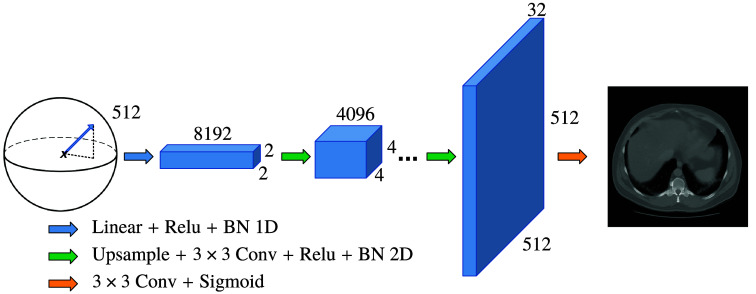
Generator DCGAN-like architecture, latent dimension (512), and final resolution (512×512) corresponding to the LDCT sub-dataset experiments.

In the presented experiments, the geometry of the decoder’s latent space is set to the unit n-sphere, similarly to state-of-the art representation learning approaches.^46^ The size of the latent space has an impact on the quality and diversity of the generated samples. A larger latent space allows for more diversity as it provides a larger space of possible inputs to the decoder, but it can also make the model more difficult to train. The size of the latent space was chosen empirically as the maximal value beyond, which no significant performance gain was observed when associated with the decoder designed for the experiments. It has been found that a good rule of thumb is to match the latent space size with the axial plane resolution of training data. Consequently, the latent space dimension is set to 320 for experiments on the LIDC dataset and 512 for the LDCT dataset. Being a reference in the literature of generative models, the deep convolutional generative adversarial network (DCGAN)^50^ architecture is used for the decoder. As illustrated in Fig. 1, the latent code first goes through a linear block and is mapped to a feature map of shape 2×2×C, with C the desired number of channels for the first convolutional layer. From layer to layer, the input is upscaled by a factor of 2 (the last layer upscale factor may vary to fit the desired output image resolution), and the number of channels is cut by half. Hence, the image resolution, the latent dimension, and C completely define the structure of the decoder. The number of input channels C is set to 8192 for both experiments on the LIDC and the LDCT sub-datasets.

cGLO optimizations are conducted with the Adam algorithm.^56^ The learning rates for the latent codes and the decoder weights are both set to $10^{-3}$ while training on CT reconstruction, i.e., the step described in Eq. (7), and respectively set to $10^{-2}$ and $10^{-4}$ when reconstructing from experimental measurements, i.e., the step detailed in Eq. (5). Although learning rates are kept constant, batch sizes are increased along specified schedules. It leads to faster training and is statistically equivalent to decreasing learning rates.^57^ Measurements are augmented by a factor of 8 for reconstructions involving parameter initialization with the training set and by factors (8, 16, and 16) for angles (9, 23, and 50) when no training data are used.

DIP, cSGM, and MCG hyper-parameters, model architecture, and other implementation details are set to what is provided by Baguer et al.,^45^ Song et al.,^25^ and Chung et al.,^26^ respectively, for their sparse-view CT experiments. The DIP network is initialized as described in Eq. (3) on a reconstruction computed with FBP. For the TV-Reg iterative scheme, the objective function being minimized for the set of viewing angles $\phi_{e}$ is

$$x^{*}=\underset{x}{\mathrm{argmin}}\;{\left\|T_{\phi_{e}}(x)-y_{\phi_{e}}\right\|}_{2}^{2}+\lambda \;\mathrm{TV}(x).$$ (8)

The objective is optimized with the Chambolle-Pock primal-dual algorithm implemented in ODL^58^ along the lines of Ref. 59. The initialization for the optimization process is set to an empty slice, i.e., an array of zeros. The weight, λ, balancing the data likelihood and the TV regularization is estimated on an external validation set of 50 slices to maximize reconstruction PSNR on that set. Likewise, the proximal step size achieving the best performances for TV-Reg is 0.75/‖L‖, with the operator norm $\left\|L\|=\|(T_{\phi_{e}},\nabla )\right\|$ as defined in Ref. 58. The estimated values of λ for every experimental configuration are presented in Table 2.

**Table 2:** Regularization weights for TV-Reg reconstructions.

Angles	LIDC 320 × 320	LDCT 512 × 512
9	$6.4\cdot 10^{-6}$	$1.6\cdot 10^{-6}$
23	$1.1\cdot 10^{-6}$	$4.8\cdot 10^{-7}$
50	$4.7\cdot 10^{-7}$	$3.3\cdot 10^{-7}$

### 3.4 Computational Complexity

Training times for cGLO and cSGM/MCG on the 2%, 10%, and 35% sub-datasets of LIDC are provided in Table 3(a). Both cSGM and MCG use the same prior training model, only inference differs. Regarding inference, the reconstruction times averaged on the five scans of the LIDC test set given 50 projections are presented in Table 3(b) for trained methods cGLO, cSGM, and MCG. The amount of training data does not influence inference computation time. Finally, the reconstruction times on the test set of 100 slices for training-free methods cGLO (0%), DIP, and TV-Reg, given 50 viewing angles, are provided in Table 3(c). All experiments were conducted on a single Nvidia A100 GPU with 40 GB of RAM.

**Table 3:** Computation times.

(a) Training time: cGLO and cSGM/MCG			
Method	2%	10%	35%
cGLO	2 h 02 min	11 h 16 min	39 h 31 min
cSGM/MCG	6 h 47 min	22 h 11 min	46 h 39 min
(b) Inference time: cGLO, cSGM, and MCG			
Method	Avg. 1 patient		
MCG	24 min 07 s		
cGLO	21 min 33 s		
cSGM	13 min 19 s		
(c) Inference time: DIP, cGLO, and TV-Reg			
Method	Tot. 100 slices	Avg. 1 slice	
DIP	8h 10 min 33 s	4 min 54 s	
cGLO	10 min 28 s	6.3 s	
TV-Reg	1 min 55 s	1.1 s	

Iterative reconstruction methods (DIP, TV-Reg, and cGLO) computation times depend on the number of iterations set for reconstruction. Consequently, cGLO and TV-Reg are firstly run for an extended amount of time, and convergence of these algorithms is defined as the point beyond, which doubling the number of iterations offers less than 1% loss reduction. Following this definition, cGLO and TV-Reg need 1000 and 600 iterations, respectively, to reach convergence. For DIP, hyperparameters proposed by Baguer et al.^45^ are used. The model is initialized on FBP for 5000 iterations, and additional $10^{4}$ iterations with forward projections are needed for reconstruction. Concerning cSGM and MCG, the iterative sampling and projection process is not directly comparable to the iterative minimization of the likelihood objective (DIP, TV-Reg, and cGLO). We set the number of iterations so that there are 800 Neural Function Evaluations (NFE), for which both models achieve the best performance. The impact of the number of NFEs on reconstruction quality is discussed in the MCG article.^26^ The training time of cGLO in Table 3(a) is quite similar to MCG/cSGM and scales almost linearly with the training sub-dataset size. As shown in Table 3(b), inference is faster when computed with cSGM, compared with cGLO and MCG. This gap is due to the back-propagation step and would be reduced by switching cGLO and MCG Pytorch^60^ implementation to the more efficient JAX^61^ implementation used by cSGM. Finally, as presented in Table 3(c), DIP inference time is substantially longer than those of cGLO and TV-Reg. DIP requires the estimation of one optimal set of U-Net weights for each slice. This optimization, averaging at 4 min and 54 s, is within the range of cGLO and TV-Reg total durations on the entire test set of 100 slices. To obtain comparable durations with DIP for the complete test set, slices would have to be reconstructed simultaneously, similar to cGLO and TV-Reg. However, running multiple network optimizations in parallel would be prohibitive in terms of GPU memory. FBP reconstructions are computed in parallel on GPU with the ASTRA toolbox,^62^ so computation time is negligible.

## 4 Results and Discussion

**Fig. 2:**
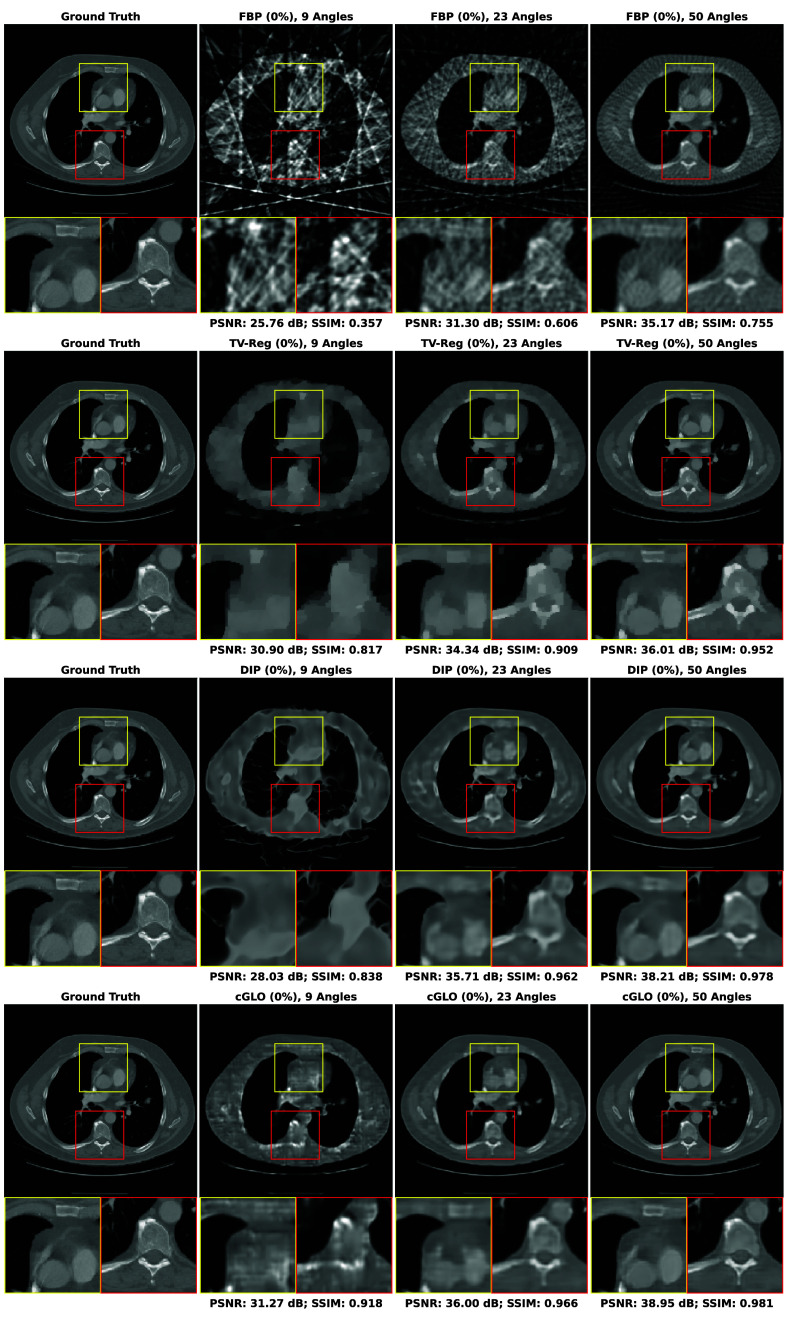
Examples of reconstructions given 9, 23, and 50 experimental viewing angles, obtained with (from top to bottom) FBP, TV-Reg, DIP, and cGLO. Reconstructions are achieved without prior unsupervised training.

**Fig. 3:**
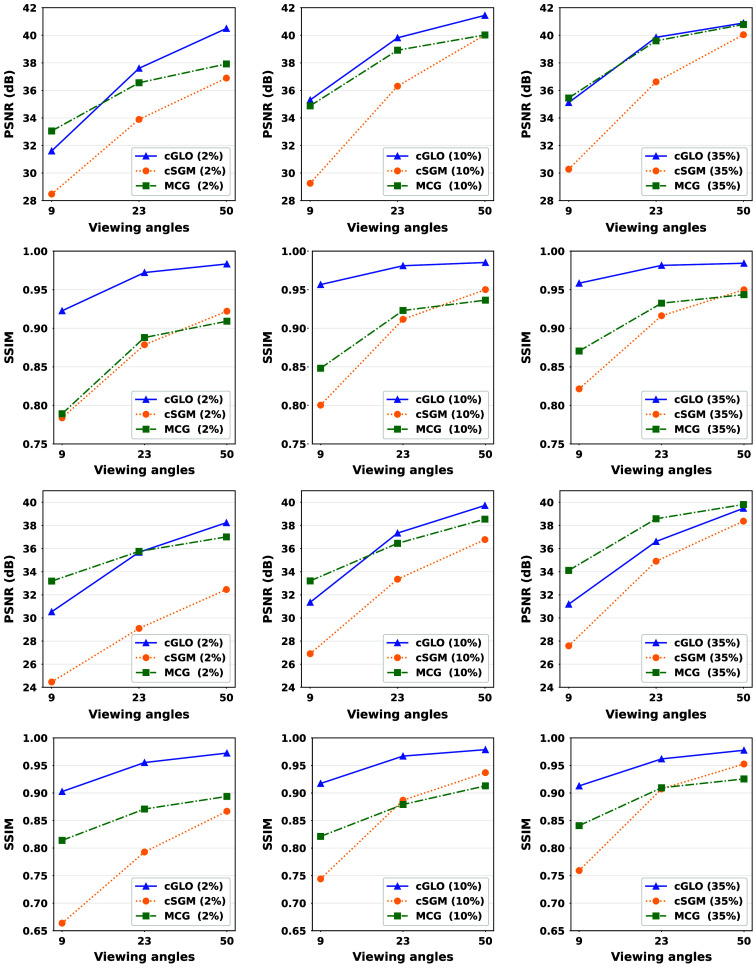
PSNR and SSIM median value curves corresponding to reconstructions of slices from the LIDC (first two rows) and the LDCT (last two rows) test sets given 9, 23, and 50 experimental viewing angles. Each column is associated with one of the 2%, 10%, and 35% training sub-datasets presented in Table 1.

**Fig. 4:**
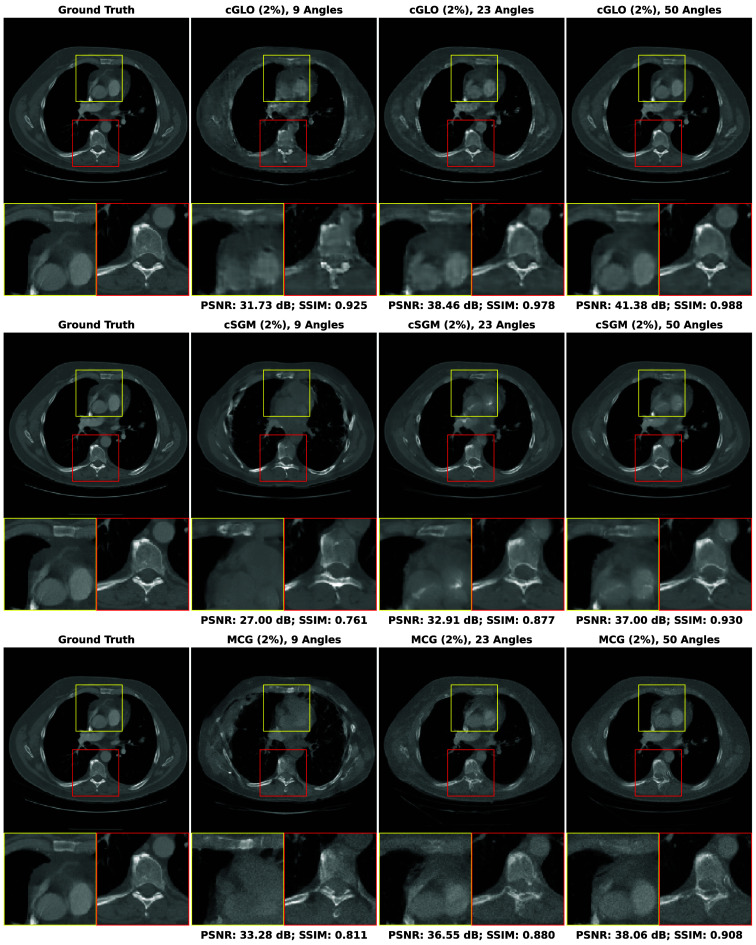
Examples of reconstructions given 9, 23, and 50 experimental viewing angles, obtained with cGLO (upper row), cSGM (middle row), and MCG (lower row). Methods are trained on the sub-dataset consisting of a 2% portion of the LIDC dataset.

In this section, our method cGLO is compared with DIP and two conventional baselines FBP and TV-Reg for reconstructions obtained without any training dataset. Regarding this specific use case, DIP currently holds state-of-the-art performance. These approaches are evaluated using quantitative pixel-wise (PSNR) and structural (SSIM) metrics, which are detailed in Table 4, alongside qualitative results consisting of examples of reconstructions from the LIDC test set and given in Fig. 2. Our method is also tested against cSGM and MCG on use cases with available training datasets of varying sizes. To the best of our knowledge, with prior unsupervised training, MCG achieves state-of-the-art reconstruction quality for sparse CT. Quantitative metrics for 9, 23, and 50 experimental viewing angles are given in Fig. 3 and detailed in Appendix D. Qualitative results consist of examples of reconstructions from cGLO, cSGM, and MCG trained on the smallest sub-dataset, i.e., a 2% portion of the LIDC dataset. These reconstructions are presented in Fig. 4. Additional examples for experimental setups corresponding to different combinations of training dataset sizes and number of viewing angles are provided in Appendix E.

**Table 4:** PSNR and SSIM median ± half interquartile range (IQR) values for reconstructions of 100 slices from the LIDC and the LDCT test datasets given 9, 23, and 50 experimental viewing angles.

Method	Angles	LIDC 320 × 320		LDCT 512 × 512	
		PSNR ↑	SSIM ↑	PSNR ↑	SSIM ↑
FBP	9	25.99±0.11	0.366±0.011	26.31±0.09	0.454±0.006
TV-Reg	9	31.15±0.20	0.821±0.005	29.93±0.23	0.777±0.002
DIP	9	28.26±0.32	0.843±0.009	27.78±0.39	0.842±0.010
cGLO	9	31.41±0.34	0.916±0.005	30.28±0.29	0.906±0.003
FBP	23	31.46±0.19	0.605±0.004	30.79±0.08	0.623±0.004
TV-Reg	23	34.39±0.22	0.911±0.003	33.17±0.79	0.892±0.002
DIP	23	35.50±0.23	0.954±0.002	33.64±0.41	0.935±0.005
cGLO	23	36.18±0.39	0.966±0.002	35.99±0.64	0.956±0.005
FBP	50	35.31±0.56	0.763±0.005	33.11±0.10	0.724±0.002
TV-Reg	50	36.21±0.20	0.953±0.002	35.87±0.80	0.937±0.001
DIP	50	37.40±0.36	0.973±0.002	36.36±0.31	0.960±0.002
cGLO	50	38.92±0.27	0.980±0.001	38.75±0.20	0.973±0.001

The best and second-best results are indicated in bold and italics for each angle and metric, respectively.

### 4.1 Without Prior Unsupervised Training

As shown in Table 4, on the use case with no training dataset available, our method cGLO outperforms DIP, TV-Reg, and FBP reconstruction methods for every set of experimental viewing angles, regarding both PSNR and SSIM metrics. Most notably given nine experimental viewing angles, cGLO offers a PSNR gain of +3.15  dB on the LIDC test set compared with DIP.

In addition, median PSNR and SSIM values reported in Table 4 indicate that the performance gap, at the advantage of cGLO over DIP, increases as the set of experimental viewing angles gets smaller. More specifically, the PSNR gap between cGLO and DIP more than doubled between experiments involving 50 (+1.52  dB) and 9 (+3.15  dB) viewing angles on the LIDC test set. In other words, our method is more robust for solving increasingly sparse CT with no prior training when compared with DIP.

Although TV-Reg offers better PSNR values than DIP for 9 (+2.89  dB) projections, DIP provides significantly higher SSIM values in this context. This corresponds to the strides that can be identified on reconstructions from TV-Reg in Fig. 2. These strides are induced by the lack of projections and could be mitigated with higher values of λ, i.e., enforcing a stronger regularization. However, this would result in lower PSNRs as the two metrics are competing objectives balanced by λ in Eq. (8).

It can also be observed in Fig. 2 on the reconstruction example given nine experimental viewing angles that, contrary to cGLO, DIP reproduces some of the deformations induced by the strong artifacts from FBP. This corroborates the larger difference between cGLO and DIP regarding the SSIM metric for this experiment (+0.073). It is a direct effect of DIP network parameters first being initialized on the FBP reconstruction. However, by skipping this initialization step, DIP reconstructions and quantitative performances would be further degraded.

### 4.2 With Prior Unsupervised Training

#### 4.2.1 Quantitative performance

As presented in Fig. 3, cGLO outperforms cSGM for all data and viewing angle configurations regarding both median PSNR and SSIM values. The largest performance gaps are observed for experiments given 9 viewing angles, with PSNRs ranging between +3.13  dB and +6.06  dB for the 2% and 10% LIDC training sub-dataset, respectively. In addition, median PSNR and especially median SSIM curves in Fig. 3 show an increasing advantage of cGLO over cSGM as the set of experimental viewing angles gets sparser and/or the training dataset gets smaller. Specifically for LIDC experiments, the lead in favor of cGLO over cSGM regarding SSIM starts at +0.034 (35% sub-dataset and 50 viewing angles) to reach +0.139 (2% sub-dataset and nine viewing angles). Furthermore, cGLO achieves higher median SSIM metrics values in all experimental setups, even in abundant data configurations with large training sub-datasets and many experimental viewing angles when compared with both cSGM and MCG. When compared with MCG on the same configurations as above, cGLO demonstrates an SSIM gain ranging from +0.041 to +0.134. Figure 3 also displays a dominance of MCG over cGLO regarding PSNR metrics on use cases with abundant data, e.g., with prior training on the 35% LDCT sub-dataset with a superiority of +0.33  dB, +1.97  dB, and +2.92  dB for PSNRs given 50, 23, and 9 viewing angles, respectively. On the smaller 2% and 10% training sub-datasets, cGLO equals or outperforms MCG regarding PSNR given experimental setups with more than 23 viewing angles. For instance, on the LIDC experiments, a gain rising from +0.9  dB (10% sub-dataset and 23 viewing angles) to +2.58 dB (2% sub-dataset and 50 viewing angles) can be observed.

The effects of training dataset sizes and viewing angles are consistent between experiments but are especially visible on the curves describing SSIM metrics for reconstructions from the LDCT test set in the last row of panels of Fig. 3. Considering one method (one color), higher vertical gaps between panels of the same row show greater dependency on training dataset sizes, whereas steeper slopes within each panel indicate a superior impact of viewing angles on performance. In this regard, cSGM is the most affected by a diminishing number of viewing angles and/or smaller training datasets. For example, when considering configurations with abundant data, e.g., the LIDC 35% sub-dataset, cSGM PSNR values plummet from 40.04 to 30.28 dB implying a −9.76  dB decrease between 50 and 9 viewing angles. As intended by its design, MCG demonstrates in the same context the most robustness (specifically regarding PSNRs) against sparser experimental setups with a −5.34 dB loss. cGLO sustains performances close to MCG with a −5.78  dB drop. Finally, our method is the most parsimonious/robust concerning training dataset sizes. Indeed, in the configuration with abundant viewing angles (50), cGLO PSNRs for LIDC experiments fall from 40.89 to 40.50 dB, i.e., a small loss of −0.39  dB when compared with MCG (−2.87 dB) and cSGM (−3.14  dB). When score models (MCG and cSGM) are trained with only 2% of the total datasets, the number of training samples is insufficient to provide a good approximation of the prior distribution. More specifically, it results in lower reconstruction quality compared with DIP in a training-free setup. In these conditions, the structural regularization induced by DIP is better, reconstruction-wise, than the poor approximation of the prior distribution by cSGM and MCG. As our method cGLO mixes both structural regularization and prior approximation during initialization, it can benefit from very small training datasets contrary to the dependency of cSGM and MCG on a large amount of training data to work properly.

The prominent points of the quantitative performance comparison among cGLO, cSGM, and MCG are the following. For every configuration, cGLO offers better reconstructions than cSGM and MCG regarding the SSIM metric and also surpasses cSGM when considering PSNR values. Further, cGLO is less dependent on training dataset sizes than MCG, which is more robust to reduced viewing angles. Consequently, when comparing PSNR values, cGLO provides better performances than MCG in scenarios with more than 23 angles and restricted access to training data, e.g., less than 10% of the LIDC dataset.

#### 4.2.2 Effect of large training datasets on cGLO

As illustrated in Fig. 3, contrary to cSGM and MCG, as the training datasets become larger, cGLO PSNR performances stagnate and even slightly worsen. More specifically for PSNRs, averaged over angle configurations, a drop is observed when cGLO is trained on the 35% rather than the 10% sub-dataset, from 38.86 to 38.62 dB for LIDC, and from 36.14 to 35.74 dB for LDCT. Given a fixed representation capacity for our decoder architecture, i.e., a fixed number of input channels C, the model becomes under-parameterized after a given training dataset size is reached. Because the model parameters and the latent codes are jointly optimized in Eq. (7) with respect to a data-likelihood objective function, the model error distribution is widely spread over the entire training dataset.^52^ Consequently, when the data variability overcomes the model capacity, each additional training example further deteriorates the average prediction quality. To demonstrate this assertion, a larger decoder with 14,336 channels in the first layer is trained on the 35% and 10% LIDC sub-dataset. This is the maximum number of channels the decoder can have before exceeding GPU memory capacity. Following the original decoder architecture, the number of channels is cut by half for each successive layer. Thanks to the larger decoder network, a significant PSNR gain averaging +0.6  dB is observed on reconstructions with prior training on the 35% sub-dataset. However, with prior training on the 10% sub-dataset, PSNR gains vary from −0.07 to +0.12  dB, which falls within the margin of error. Ideally, the model capacity should be tuned to best fit each experimental setup. However, the experiments realized in this paper demonstrate that even with a fixed decoder architecture, cGLO produces superior reconstructions for multiple experimental setups.

#### 4.2.3 Reconstruction structural quality

Figure 4 compares reconstructions computed with cGLO, cSGM, and MCG from the LIDC test dataset. Reconstruction quality clearly degrades as the number of experimental viewing angles diminishes. The type of degradation, however, differs between cGLO and the score-based methods cSGM and MCG. Although cGLO reconstructions lack sharp details and get noisier for very sparse CT or when trained on very small datasets, cSGM and MCG tend to alter the structural integrity of the slices. This observation corroborates and explains the total dominance of cGLO over all the methods when comparing median SSIM curves rather than median PSNR curves in Fig. 3.

The fact that cSGM/MCG and cGLO yield different reconstruction structures for more difficult experimental setups can be explained by the way each model employs experimental data. cSGM uses a proximal optimization step to mix experimental data with unconditioned samples. This leads to a cost function expressing a balance between data likelihood and prior, which only enforces a loose constraint on the conformity of the proposed reconstruction to experimental data. When the number of viewing angles diminishes, there are increasing structural deformations as shown in Fig. 4. The MCG correction step prevents the inference mechanism from deviating from data consistency for sparse experimental setups. However, when trained on insufficient data quantities, the assumption that the score model approximates the data manifold may not hold anymore. On the contrary, the cost function of cGLO, described in Eq. (5), enforces a strict constraint on experimental data. As seen in Fig. 4, a decrease in viewing angles leads to degraded reconstructions with more noise and less details at nine viewing angles for example. However, contrary to cSGM and MCG, cGLO does not add non-existent structures to reconstructions, such as blood vessels or bones. These additions of local patterns learned during training are specific to generative models. They are different from artifacts produced with training-free methods, as those do not depend on a learned prior, i.e., on the nature of images being reconstructed.

### 4.3 Acquisition Setup

The discrete forward operator $T_{\phi_{e}}$ involved in the sparse-view CT experiments conducted in this paper, and described in Sec. 3.2, follows an undersampled parallel-beam geometry without noise insertion. The choices leading to this acquisition setup and the limitations they imply are discussed here.

#### 4.3.1 Undersampling

When undersampling projections, the same subset of views is used for each slice. Taking different subsets of views for each slice would be an unfair advantage benefiting our method cGLO, which performs joint reconstructions over competing methods computing independent reconstructions. Indeed, the error of one slice is back-propagated through the shared decoder and impacts other slice reconstructions. In that sense, we could also say that information accessible for one slice is usable to reconstruct other slices. Consequently, if each slice has a different subset of views our method has indirect access to a larger subset of views. This effect is further examined in Appendix B.

#### 4.3.2 Parallel-beam

Parallel-beam acquisition geometry was naturally chosen because every competing method we compare our approach to was tested under this geometry in their original papers: cSGM,^25^ MCG,^26^ and DIP.^45^ These methods rely on 2D generative models (cSGM, MCG) or a 2D U-Net architecture for reparameterization (DIP). Consequently, they require a slice-independent forward operator (e.g., parallel-beam or fan-beam) due to their objective functions assuming separate contributions between slices, i.e., reconstructing slices independently. However, many practical use cases require 3D acquisition geometries, e.g., cone-beam.

The DIP approach has been extended by Ref. 63 to reconstruct volumes directly with a network architecture using 3D convolutions, as opposed to the stacking of slices in the original strategy.^45^ Although tested on limited-angle CT with a parallel-beam configuration, the method could be applied under 3D acquisition setups.

Extending DMs (cSGM, MCG), and generative models in general, to 3D inverse problems remains a difficult task due to extremely high memory and computational cost associated with learning the prior distribution of 3D objects. Alternatively, a recent approach proposed by Ref. 64 adapts the 2D reconstruction process of MCG^26^ by imposing a TV penalization along the vertical axis orthogonal to the plane of the slice through an intermediate optimization step. This strategy demonstrates better vertical coherence of slices, leading to improved 3D reconstructions compared with 2D slice stacking. However, being based on the MCG 2D reconstruction approach, it is still restricted to slice-independent forward operators.

Although not demonstrated in this manuscript through dedicated experiments, we would like to point out that cGLO could also theoretically be extended to 3D acquisition geometries while having a simple 2D decoder architecture. As the error made on one slice changes the estimation of other slices, our method does not require a forward operator to treat each slice independently. The results presented in this paper under parallel-beam geometry cannot be extrapolated to fully 3D CT geometries, and a rigorous estimation of cGLO performances in these configurations should be conducted in future studies to further assess the applicability of the method in such clinical contexts.

#### 4.3.3 Measurement degradations

Finally, in our approach, the same discrete forward operator is used for the ground-truth projections and the reconstruction process. It constitutes an inverse crime, as defined in Ref. 65. Good reconstruction quality, obtained under an inverse crime approach may stem from the specificity of the discrete acquisition geometry. Further, the sensitivity of the method to noise or other small variations occurring in real projection data is not estimated. An experiment in Appendix C, in which reconstructions are computed from degraded measurements, illustrates the robustness of our approach to cGLO.

## 5 Conclusion

In this work, we presented cGLO, a unsupervised method to solve IIPs built upon the general framework of GLO.^46^ Contrary to supervised strategies, our method does not require a fixed experimental setup. Analogously to DIP,^45^ our strategy benefits from the structural prior induced by convolutional decoders without any preceding training. Furthermore, cGLO takes advantage of a shared objective function by reconstructing multiple slices simultaneously at examination time and may be initialized beforehand on an unsupervised training dataset of any size to improve reconstruction quality. In other words, our approach is a very flexible plug-and-play reconstruction technique that can be used with or without unsupervised training data and without any assumption on the IIP at hand.

cGLO was tested on full-dose sparse view CT, which is a widely studied reconstruction problem arising in medical imaging and compared with the current state-of-the-art approaches, MCG^26^ for unsupervised cases and DIP for cases with no prior training. Experiments conducted in this paper cover a wide range of realistic setups with varying amounts of available training data and experimental viewing angles. Quantitative results regarding pixel-wise metric (PSNR) demonstrate that cGLO is a parsimonious and robust reconstruction method as it offers similar to better performances when compared with MCG on small data regimes with sufficient viewing angles and surpasses DIP in every experimental setup. Moreover, for the entire set of conducted experiments, cGLO offers the best performances with respect to the structural metric (SSIM). Indeed, reconstruction examples illustrate that cGLO exhibits a propensity to preserve the structural integrity of reconstructions and it avoids the inclusion of learned local patterns, even for very ill-posed IIPs, thanks to its straightforward conditioning to experimental measurements.

Although the performance of cGLO has been tested on a tomographic reconstruction problem, the framework developed in this article can be readily applied to other ill-posed IIPs, such as MRI reconstruction from a sparsely sampled k-space. Furthermore, because the cGLO approach only requires the knowledge of the forward operator, it could be extended to solve non-linear IIPs. One of the main interests of cGLO is that it does not require any backward operator, such as FBP for tomographic reconstruction. Consequently, it is a method of choice for IIPs where such operators do not exist, such as multi-material CT reconstruction. Future work will explore the application of cGLO to such IIPs. Because cGLO efficiently learns correlations between the output channels of its decoder network, further developments will also focus on multi-task applications, such as jointly learning reconstruction and segmentation.

## 6 Appendix A: Effect of the Interpolation Factor

**Fig. 5:**
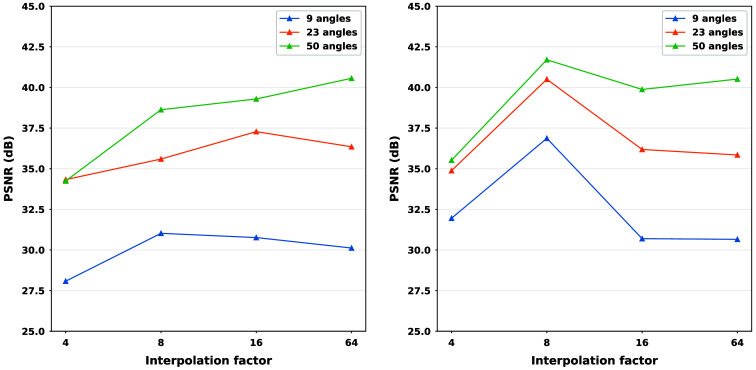
Median PSNR values for reconstructions of slices from the LIDC test set given: 9, 23, and 50 experimental viewing angles. They are obtained with cGLO without any training dataset (left) and trained on the 35% portion of the LIDC dataset (right).

In this study, the impact of the number of experimental profiles provided during the optimization process described in Eq. (5) on reconstruction performances is examined. For this purpose, five independent reconstructions, one for each patient of the LIDC test set, are conducted for several interpolation factors: 4, 8, 16, and 64. Independently for each patient, the augmentation is achieved by inserting new artificial profiles computed by linear interpolation between the existing experimental profiles along the axis orthogonal to the plane of the slice. The interpolation factor corresponds to the number by which the initial profile quantity is multiplied in the process. Reconstruction quality is evaluated using median PSNR values over the entire LIDC test set for varying experimental setups with: 9, 23, and 50 viewing angles. Reconstructions computed using artificial profiles are discarded. These quantitative metrics are given in Fig. 5 for two experiments, one with no prior training and the other with the decoder trained on the sub-dataset consisting of a 35% portion of the LIDC dataset.

The results presented in Fig. 5 indicate that the interpolation factor has a significant impact on reconstruction quality by inducing great variations (several dBs) for each curve, i.e., each experimental setup. Concerning experiments with prior unsupervised training, our model displays a consistent behavior across angles with peak performance for a factor of 8. In addition, a greater performance gap benefiting higher numbers of viewing angles can be observed for increasing interpolation factors both for experiments with and without prior training.

The interpolation factor used in our method can be seen as a hyper-parameter balancing the learned prior in Eq. (7), and the data-likelihood enforcing experimental profile consistency during the reconstruction task at examination time in Eq. (5). Indeed, for sufficiently important interpolation factors (>16), performances with prior training become similar to those without any training. It implies that beyond a given interpolation factor value, decoder initialization, i.e., prior information learned on the unsupervised training sub-dataset, is completely overwritten by the second optimization.

Consequently, the interpolation factor should be adapted for each use case, with higher values for experimental setups involving more viewing angles and a potential upper limit depending on the quantity of training data at hand.

## 7 Appendix B: Effect of Random Subset of Views

**Fig. 6:**
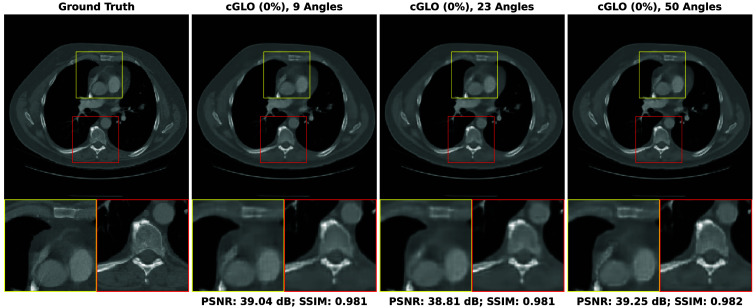
Examples of reconstructions of slices from the LIDC test set given 9, 23, and 50 experimental viewing angles with random starts and no prior training.

To demonstrate the unfair advantage our joint reconstruction method cGLO would have over the independent reconstruction method, and to illustrate this interesting property of shared knowledge between slices, a simple additional experiment is conducted. The following experiment is the same as cGLO (0%), i.e., without prior training, except that the start of the subset of equidistant angles is not set at 0 deg but sampled uniformly for each slice between 0 deg and 180  deg/N, with N the number of angles. For instance, with three viewing angles, the random subset would be {0  deg+a,60  deg+a,120  deg+a} with a sampled uniformly between 0 deg and 60 deg. That way, the exact partitioning and range of angles are preserved and it is ensured that views are not mirrored above 180 deg. The PSNR and SSIM metrics are averaged on the same 100 slices test set used for FBP, DIP, and TV-Reg. The results are presented in Table 5 and reconstruction examples are given in Fig. 6.

**Table 5:** PSNR and SSIM median ± half interquartile range (IQR) values for reconstructions of slices from the LIDC test dataset given 9, 23, and 50 viewing angles subsets with random starts.

Method	Angles	LIDC 320 × 320	
		PSNR ↑	SSIM ↑
cGLO (0%) (random starts)	9	39.05±0.31	0.981±0.001
	23	38.89±0.29	0.980±0.001
	50	39.41±0.27	0.982±0.001

As expected, both quantitative and qualitative results indicate that, if each slice has a different subset of views, reconstruction quality does not vary with the number of viewing angles. In other words, the amount of accessible information is the same given 9, 23, and 50 viewing angles.

## 8 Appendix C: Effect of Measurement Degradations

In this experiment, the impact of natural degradations, occurring on clinical projection data, on reconstruction quality is estimated. First, our method cGLO is trained on the 35% LIDC sub-dataset. The largest sub-dataset was chosen to avoid mixing multiple causes for performance loss, small training datasets, and measurement degradations. Reconstructions are computed given 9, 23, and 50 simulated and artificially degraded projections acquired under a parallel-beam geometry acquisition. The discrete operator used in the optimization process is the same as previous experiments involving the inverse crime. Simulated projections used as experimental ground-truths are prepared by first upsampling the slices with bilinear interpolation, from a resolution of 320×320 to 1000×1000  pixels. The same detector geometry is used, i.e., 453 equidistant detector bins spanning the slice diagonally. Then, a small noise following the Poisson probability distribution Poisson(λ) is added to projections $y=T_{\phi_{e}}(x)$ with resolution $m=453\times \phi_{e}$, such that

$$y^{\delta }∼\text{Poisson}(N_{0}e^{-y}),$$ (9)

with $y^{\delta }$ the random variable corresponding to degraded observations according to a mean photon count per detector bin without attenuation $N_{0}=10^{5}$ and the Poisson(λ) distribution defined as

$$P(X=k)=\frac{\lambda^{k}e^{-\lambda }}{k!},\;\text{for}\;k\in [0,\ldots ,N_{0}].$$ (10)

**Fig. 7:**
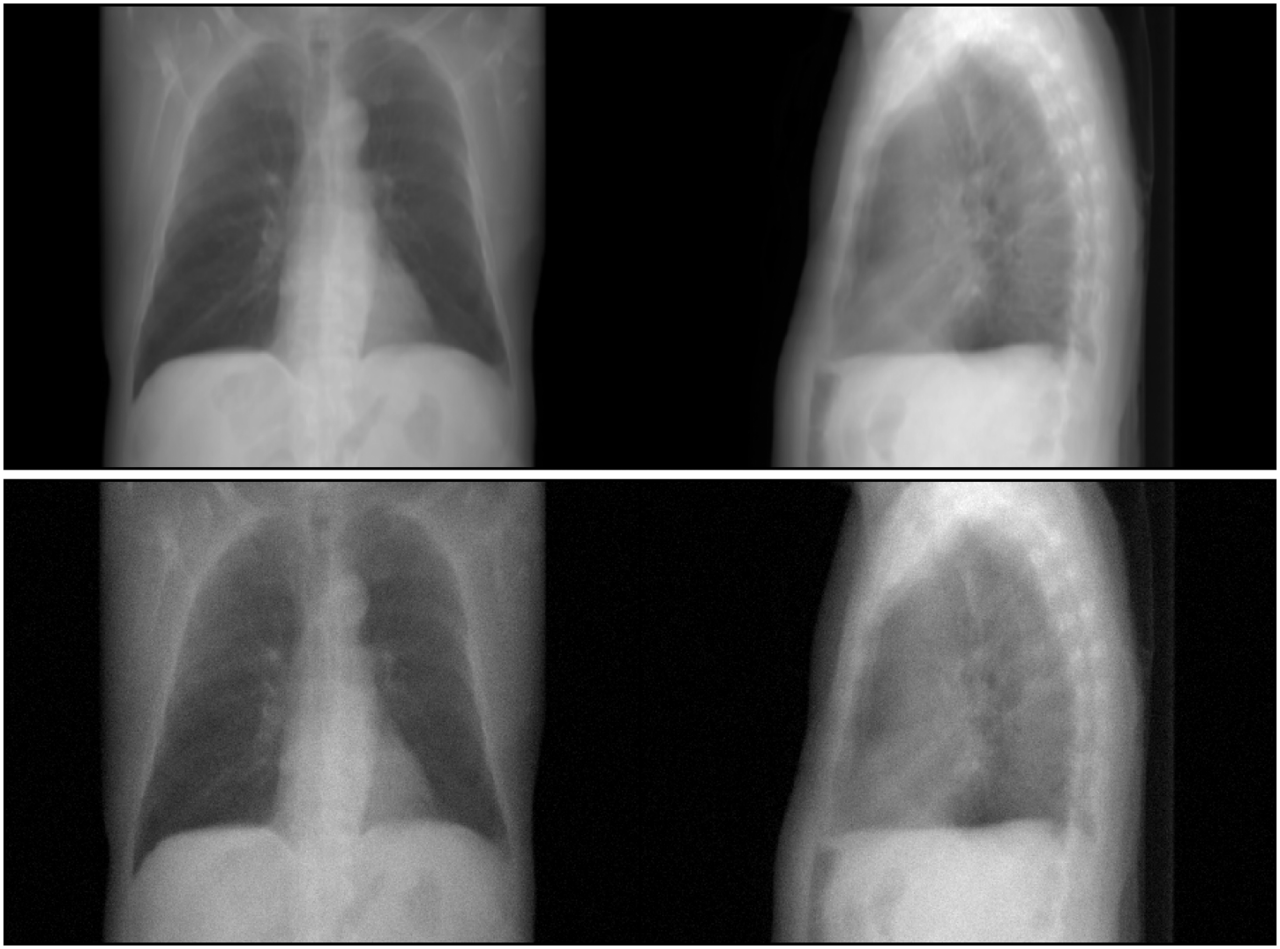
Simulations of the face (left) and side (right) radiographic images of a patient’s lungs. The images are computed under parallel beam geometry without degradation (top) and with interpolation and added Poisson noise (bottom).

The parameter $\lambda =N_{0}e^{-y}$ is homogeneous to the observations y, i.e., $\lambda \in R^{m}$, so Poisson(λ) denotes m independent and identically Poisson distributed pixels with parameters $\lambda_{1},\ldots ,\lambda_{m}$ respectively. A log-transformation may be applied as a preprocessing step^66^ to obtain the linear model $y^{\delta }=y+\delta$, but then, the random observations $y^{\delta }$ do not follow a Poisson distribution anymore. In this experiment, we do not use a log transformation of the noisy observations. Examples of resulting projections are presented in Fig. 7.

To accommodate the maximum a posteriori formulation presented in Eq. (5) to the Poisson noise, the squared L2-norm $\left(\|\cdot {\|}_{2}^{2}\right)$ is replaced by the Poisson negative log-likelihood (PNLL) loss, with the constant Stirling factorial approximation of degraded observations $C(y^{\delta })\in R^{m}$, as implemented in Pytorch

PNLLZ,θ(y^,yδ)=y^−yδ log(y^)+C(yδ),where  y^=N0 exp[−Tϕe∘fθ(Z)]. (11)

**Fig. 8:**
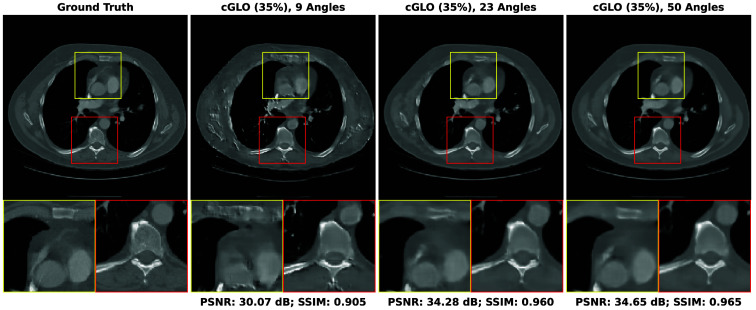
Examples of reconstructions of slices from the LIDC test set given: 9, 23, and 50 experimental viewing angles. They are obtained with cGLO trained on the 35% LIDC sub-dataset.

A Gaussian approximation of the Poisson noise is commonly used for CT reconstruction objectives^67^ because the negative log-likelihood is reduced to a weighted L2 loss, which generally facilitates the optimization process. In the experiments conducted here, the straightforward PNLL loss is preferred since it is readily accessible in Pytorch and avoids an additional approximation, benefitting results interpretation. The metrics given degraded projections are averaged on the separate reconstructions of the five patients of the LIDC test set. Results are presented in Table 6 and corresponding reconstruction examples are illustrated in Fig. 8.

**Table 6:** PSNR and SSIM median ± half interquartile range (IQR) values for reconstructions of slices from the LIDC test dataset given 9, 23, and 50 degraded projections.

Method	Angles	LIDC 320 × 320	
		PSNR ↑	SSIM ↑
cGLO (35%) (degraded measurements)	9	31.13±1.82	0.922±0.032
	23	34.05±1.58	0.955±0.020
	50	34.65±1.77	0.963±0.013

As expected, the results worsen when compared with those obtained with the inverse crime approach due to the higher lower bound in the error to the projections corresponding to the magnitude of the added Poisson noise. Quantitatively, the loss in reconstruction quality stays in the same range over different numbers of viewing angles, with PSNR losses varying from −3.98  dB at 9 angles to −5.80  dB at 23 angles. Qualitatively, no major artifacts or alteration appear in reconstructions presented in Fig. 8 when compared with those given in Fig. 11. This suggests that our method cGLO is not overly sensitive to noise and that reconstructions presented under the inverse crime approach are not the results of the specific discretization of our forward operator.

## 9 Appendix D: Additional Results Tables

In this section, the quantitative results presented in Fig. 3 are detailed. The results consist of PSNR and SSIM median values and half IQR for reconstructions of the LIDC and LDCT test sets with our method cGLO, cSGM,^25^ and MCG^26^ with training sub-datasets of varying sizes as given in Table 1. Results for each method and sub-dataset are presented for nine viewing angles in 7a, 23 viewing angles in 7b, and 50 viewing angles in 7c.

**Table 7:** PSNR and SSIM median ± half interquartile range (IQR) values for reconstructions of slices from the LIDC and the LDCT test datasets given: (a) 9, (b) 23, and (c) 50 viewing angles.

Method	Data	LIDC 320 × 320		LDCT 512 × 512	
		PSNR ↑	SSIM ↑	PSNR ↑	SSIM ↑
(a) 9 viewing angles					
cSGM	2%	28.47±1.04	0.784±0.027	24.46±1.10	0.664±0.059
MCG	2%	33.05±1.35	0.789±0.020	33.19±1.15	0.814±0.033
cGLO	2%	31.60±1.30	0.923±0.016	30.53±0.98	0.903±0.014
cSGM	10%	29.25±1.00	0.800±0.020	26.91±1.08	0.744±0.041
MCG	10%	34.88±1.12	0.848±0.013	33.20±1.07	0.821±0.024
cGLO	10%	35.31±0.94	0.957±0.008	31.35±0.99	0.917±0.017
cSGM	35%	30.28±1.13	0.821±0.031	27.59±1.31	0.759±0.038
MCG	35%	35.45±1.19	0.871±0.017	34.11±1.11	0.841±0.019
cGLO	35%	35.11±0.97	0.958±0.006	31.19±1.08	0.913±0.016
(b) 23 viewing angles					
cSGM	2%	33.89±1.01	0.879±0.019	29.09±0.61	0.793±0.018
MCG	2%	36.55±1.23	0.888±0.012	35.76±1.41	0.871±0.015
cGLO	2%	37.60±1.67	0.972±0.009	35.68±1.03	0.955±0.010
cSGM	10%	36.31±0.79	0.912±0.015	33.35±1.02	0.887±0.017
MCG	10%	38.92±0.92	0.923±0.010	36.45±1.28	0.879±0.013
cGLO	10%	39.82±1.26	0.981±0.006	37.34±1.40	0.967±0.010
cSGM	35%	36.62±1.61	0.916±0.024	34.90±1.08	0.907±0.015
MCG	35%	39.60±1.17	0.933±0.014	38.58±1.37	0.910±0.013
cGLO	35%	39.85±1.19	0.982±0.005	36.61±1.45	0.962±0.010
(c) 50 viewing angles					
cSGM	2%	36.90±0.83	0.922±0.016	32.46±0.56	0.867±0.015
MCG	2%	37.92±0.85	0.909±0.008	37.01±1.46	0.894±0.009
cGLO	2%	40.50±1.56	0.983±0.007	38.24±1.05	0.972±0.007
cSGM	10%	40.03±1.05	0.950±0.016	36.78±0.96	0.937±0.013
MCG	10%	40.02±1.10	0.936±0.009	38.55±1.38	0.913±0.011
cGLO	10%	41.45±1.53	0.985±0.006	39.73±1.31	0.979±0.007
cSGM	35%	40.04±1.45	0.950±0.019	38.37±1.05	0.953±0.010
MCG	35%	40.79±1.21	0.943±0.008	39.81±1.42	0.939±0.010
cGLO	35%	40.89±1.38	0.984±0.006	39.48±1.43	0.978±0.008

The best and second-best results are indicated in bold and italics for each sub-dataset and metric, respectively.

## 10 Appendix E: Additional Reconstruction Examples

**Fig. 9:**
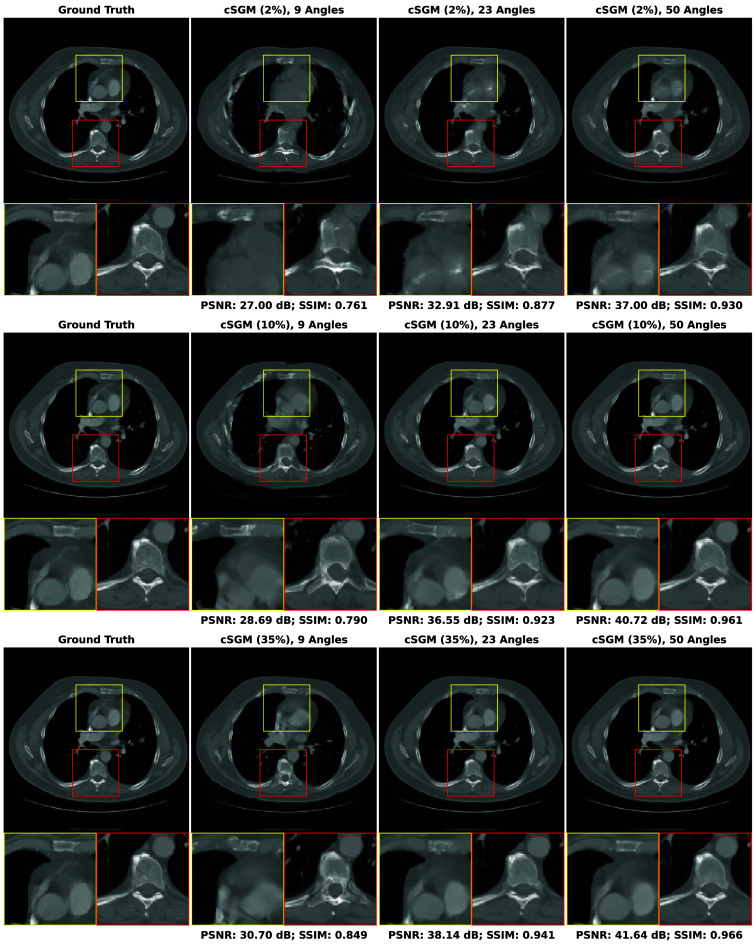
Examples of reconstructions of slices from the LIDC test set given: 9, 23, and 50 experimental viewing angles. They are obtained with cSGM trained on the 2%, 10%, and 35% LIDC sub-datasets.

**Fig. 10:**
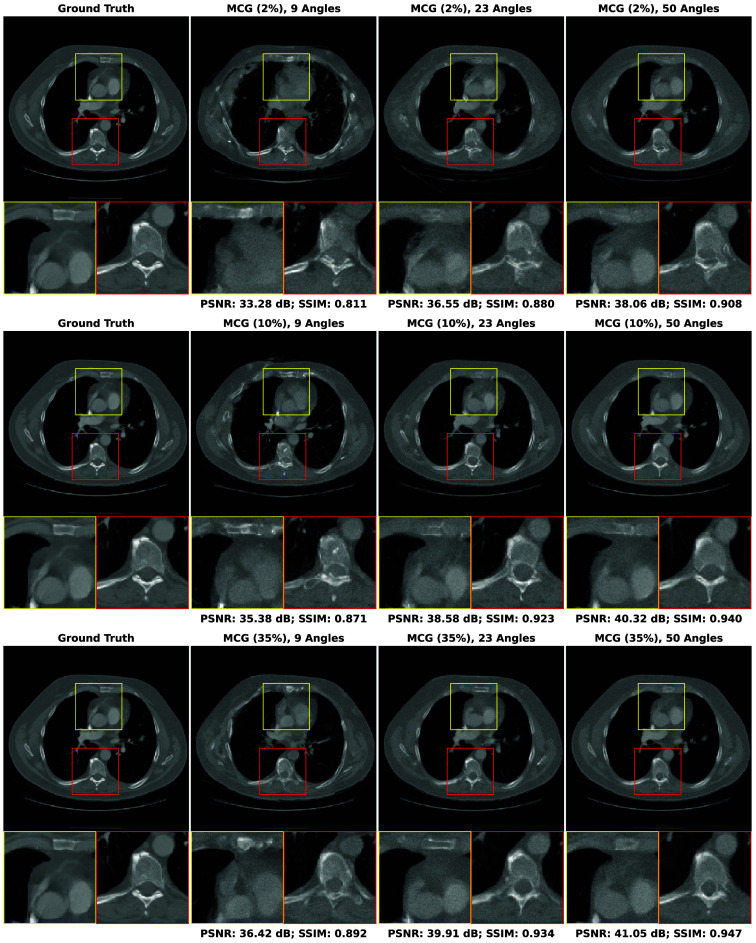
Examples of reconstructions of slices from the LIDC test set given: 9, 23, and 50 experimental viewing angles. They are obtained with MCG trained on the 2%, 10%, and 35% LIDC sub-datasets.

**Fig. 11:**
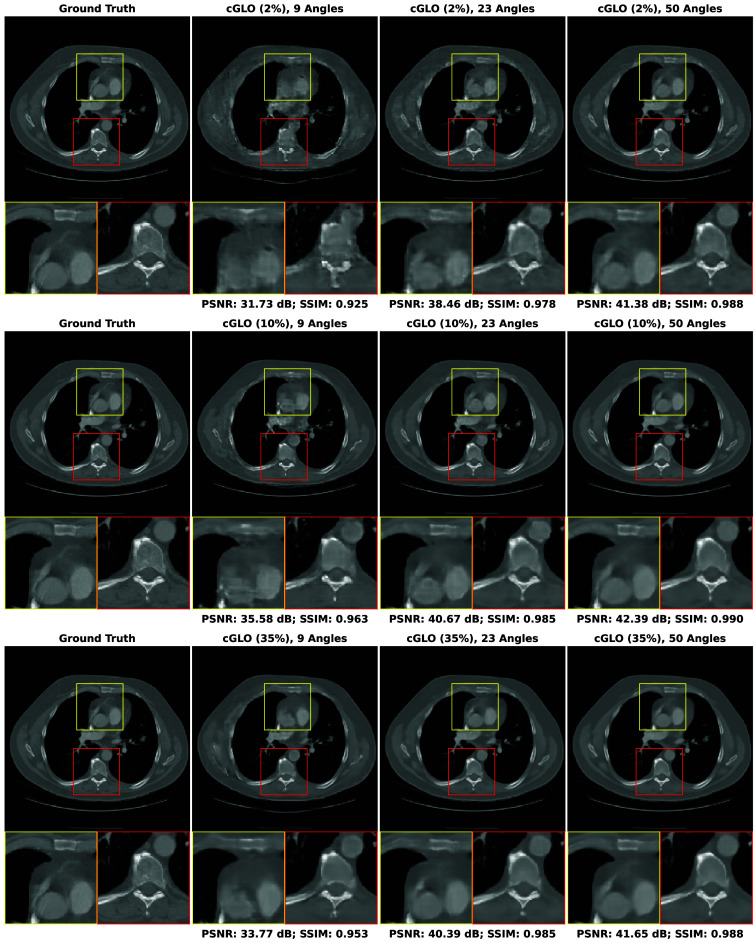
Examples of reconstructions of slices from the LIDC test set given: 9, 23, and 50 experimental viewing angles. They are obtained with cGLO trained on the 2%, 10%, and 35% LIDC sub-datasets.

The qualitative results presented in this Appendix complete the reconstruction examples given in Fig. 4 by covering experiments on the LIDC test set given 9, 23, and 50 viewing angles for each LIDC training sub-dataset in Table 1(a). Each figure displays every reconstruction example for one method. Figures are organized as follows: Fig. 9 for cSGM,^25^ Fig. 10 for MCG,^26^ and Fig. 11 for cGLO.

## Data Availability

The code to reproduce the experiments conducted in this paper is available on GitHub (github.com/tbraure/Conditioning-Generative-Latent-Optimization-for-Sparse-View-CT-Image-Reconstruction). The Lung Image Database Consortium image collection and the low-dose CT image and projection dataset are publicly available on The Cancer Imaging Archive platform (www.cancerimagingarchive.net).
